# Application of the London Atlas Software App 2nd edition for the radiographic assessment of dental development

**DOI:** 10.4317/jced.59974

**Published:** 2022-11-01

**Authors:** Erica Dalben, Juliano Bueno, Yasmin Fonseca, Scheila Mânica, Monikelly Nascimento, Ademir Franco

**Affiliations:** 1 Division of Forensic Dentistry, Faculdade São Leopoldo Mandic, Campinas, Brazil; 2 Division of Oral Radiology, Faculdade São Leopoldo Mandic, Campinas, Brazil; 3 Centre of Forensic and Legal Medicine and Dentistry, University of Dundee, UK; 4 Department of Therapeutic Stomatology, Sechenov University, Moscow, Russia

## Abstract

**Background:**

Assessing the dental development of children and adolescents is an important part of treatment planning. The radiographic visualization of dental developmental stages prior to age estimation is currently feasible by means of digital software apps. Testing the existing software tools is necessary to safeguard application in practice. This study applied the London Atlas Software App 2nd edition™ for dental age estimation in Brazilian children.

**Material and Methods:**

The software was applied to 1.104 digital panoramic radiographs of females (n = 509) and males (n = 595) with ages between 6 and 15.99 years (mean = 10.88 ± 2.84 years). The sample included at least 100 individuals similarly distributed based on sex within 10 age categories of one year each (6├ 15.99) years. Metrics of errors were quantified between the estimated (EA) and chronological (CA) ages.

**Results:**

The mean absolute errors among females and males were 0.56 and 0.60 years, respectively (overall = 0.58 years for the combined sample). The lower error values were observed in the age interval of 6 ├ 9.99 years. Error values above one year were detected in older age intervals (10 ├ 15.99 years). Statistically significant differences in dental development were not observed between females and males (*p*> 0.05).

**Conclusions:**

The London Atlas Software App 2nd edition led to specific error rates that can be acceptable for case-specific clinical applications. In the forensic field, caution is advised if the application is planned in the transition between late childhood and early adolescence – when third molars play a major role among the scarce developing teeth.

** Key words:**Brazil, children, dental age estimation, forensic dentistry, radiology.

## Introduction

Published as an article 12 years ago (1), the London Atlas gained popularity worldwide as a method for dental age estimation (2-8). Originally, the London Atlas was based on intrauterine (n = 72) and post-natal (n = 104) skeletal specimens of the Royal College of Surgeons of England, as well as on a dataset of radiographs from the living (n = 528 equally distributed per sex). The method presents 23 schematic drawings of the deciduous and permanent teeth from the age of 28 weeks in utero to the age of 15.5 years (1). Eight additional drawings are specifically used to illustrate the third molar development in the age interval of 16-23 years (drawings starting at 16.5 years and finishing at 23.5 years) (1). Dental development and root resorption were addressed according to the system of Moorrees *et al*. (1963a) (9) and Moorrees *et al*. (1963b) (10) respectively; while alveolar eruption was addressed following a modified system of Bengston (1935) (11). In 2021, the interactive software version of the London Atlas was updated into a 2nd edition (https://www.qmul.ac.uk/dentistry/atlas/software-app/). Ongoing curation is accomplished by the Institute of Dentistry – Faculty of Medicine and Dentistry, Queen Mary University of London, and the software is available in 22 languages.

Because the London Atlas includes developmental stages of teeth in utero, during childhood, adolescence, and early adulthood, applications can be forensic or clinical. Dental age estimation in forensic cases may include adoption, human trafficking, asylum seekers, legal imputation, and identification of the deceased (12-18). Clinical applications include treatment planning and timing of therapeutic procedures in Orthodontics, Paediatric Dentistry, and Special Care in Dentistry, among other fields (19-22). In general, the studies that subsequently tested the application of the London Atlas for international practice sampled panoramic radiographs (2-8) – as these are retrospectively obtained from existing image databases in observational studies.

The current literature shows important limitations in the sampling process. These limitations heavily rely on sample size, and especially on the distribution of radiographs based on age and sex. Studies in India (6), Iran (3), and Thailand (8), for instance, sampled only 335, 339, and 395 individuals below the age of 16 years.

Other studies, opted for larger age intervals such as the studies with the Brazilian (with 288 radiographs) (5) and Portuguese (with 736 radiographs) (2) populations, which included adolescents and young adults (and included the analysis of third molar formation). Among the few studies that accomplished an equal distribution of females and males, the one performed by Correia *et al*. (2020) (4) had the largest and more balanced sample. The study, however, was restricted to Brazilian adolescents (age 16-21 years) and was designed for binary outcomes (i.e. minor or adult) around the legal age threshold of 18 years – targeting forensic application based on legal majority.

Despite the existing studies with the London Atlas in the Brazilian population, no study has been performed with the updated 2021’s Software App (2nd edition). This study aimed to compare the chronological age of Brazilian female and male children from with their estimated age obtained with the London Atlas Software App 2nd edition.

## Material and Methods

-Study design and ethical aspects

This study consisted of an observational, analytical, cross-sectional study. Hence, the structural written components followed The Strengthening the Reporting of Observational Studies in Epidemiology (STROBE) Statement available within the Enhancing the Quality and Transparency Of health Research (EQUATOR) checklists. The study followed the World Medical Association’s Declaration of Helsinki (2013). Thus, patients were not exposed to ionizing radiation for research purposes. All the radiographs used in this study were collected retrospectively from an existing and private image database. Institutional ethical approval for human research was obtained (protocol: 49906221.2.0000.5374).

-Participants and settings

The sample of the present study consisted of panoramic radiographs (n = 1.104) of female (n = 509, 46.11%) and male (n = 595, 53.89%) Brazilian children with age between 6 and 15.99 years (females’ mean age = 10.95 ± 2.84 years; males’ mean age = 10.83 ± 2.84 years).

The radiographs were retrospectively collected from an existing image database in Central-West Brazil. All the radiographs were obtained from children under dental treatment between the years 2012 and 2022. The inclusion criteria consisted of children of Brazilian nationality, with ages between 6 and 15.99 years, and at least one panoramic radiograph in the image database. The exclusion criteria consisted of panoramic radiographs with missing, decayed, restored, or obturated (root canal) teeth in the third quadrant (mandibular left side), presence of visible bone lesions, surgical appliances in the mandible, deformation of maxillofacial bones, and visible dental anomalies, images with poor image quality, and missing data about the date of image acquisition, and patient’s date of birth and sex. A minimum sample size of 440 individuals was estimated using a single mean estimation with a standard deviation of 14 units in maturity score and precision of 5 units. With more representatives to be allocated based on sex and age, the sample collected in this study was balanced to distribute females and males per age category of one year each (Table 1). A recent systematic literature review (15) with dental age estimation methods applied to Brazilian children showed that studies with a similar sample size led to adequate effects among females and males. The obtained panoramic radiographs were imported to GNU Image Manipulation software (GIMP Team, International) for visualization on a 15” computer screen. The software package enabled magnification up to 100% and adjustments of brightness and contrast.

**Table 1:**
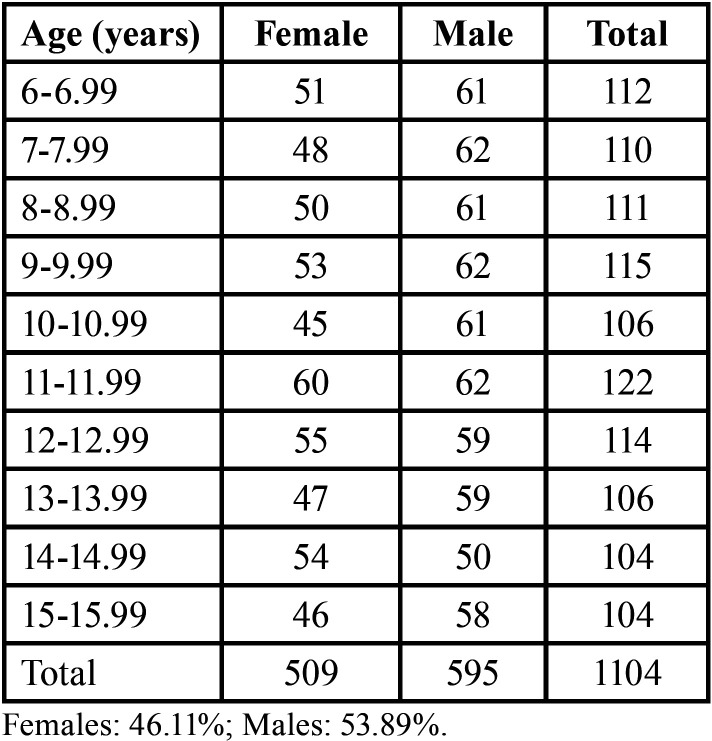
Sample distribution based on age and sex.

-Data source and variables

The variables considered in this study were sex (I), and the chronological (II) and estimated (III) ages of the individuals. Sex was registered from each panoramic radiograph. The chronological age was obtained by subtracting the date of birth from the date of radiographic image acquisition of each individual. For a deeper analysis of the method, the chronological age was converted into categorical data by dividing the sample into 10 age groups: 6├ 6.99 years; 7├ 7.99 years; 8├ 8.99 years; 9├ 9.99 years; 10├ 10.99 years; 11├ 11.99 years; 12├ 12.99 years; 13├ 13.99 years; 14├ 14.99 years; and 15├ 15.99 years.

**Figure 1:**
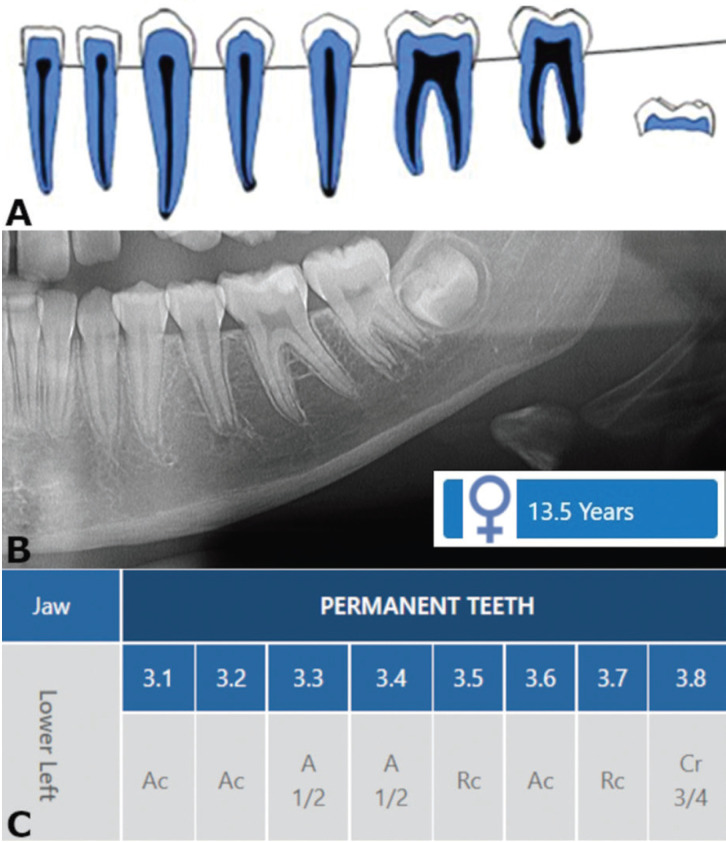
Illustration of the radiographic analysis performed for dental age estimation using the London Atlas Software Application 2nd edition. Caption: Representation of the developmental stages illustrated using London Atlas style (A) after radiographic visualization (B) of the mandibular left permanent teeth (including third molar). In “data entry”, the interactive tool enables the classification of the teeth following the staging system proposed by Moorrees *et al*. (9) (C). Prior to the classification process, the operator can select the sex (male, female or unknown), dentition (deciduous or permanent), quadrant of the teeth under analysis (upper right, upper left, lower left, and lower right), and the notation system to be used (anthropological, International Dental Federation, Palmer’s or Universal).

The estimated age was obtained through the application of the London Atlas via Software App 2nd edition. Image analysis was performed in a dimmed room under standard viewing conditions, and no more than 25 radiographs were analyzed per day to avoid visual fatigue. Using the data entry function, the sex of each individual was selected, followed by the selection of permanent dentition, mandibular left quadrant, and notation system of the International Dental Federation (FDI). In each panoramic radiograph, the permanent mandibular teeth from the third quadrant were analyzed. These teeth were classified into developmental stages according to the system of crown, root, and apex formation proposed by Moorrees *et al*. (1963a) (9) (Fig. 1). In this process, the third molar was included to add as much as possible age-related developmental information in the process of age estimation. On the contrary, we did not add eruption-related information to the age estimation process because, in children, developmental parameters generally lead to more reliable estimates compared to eruption. It must be noted that both the classification system proposed by Moorrees *et al*. (1963a) (9) and the inherent interactive software application of London Atlas are listed among the dental age estimation methods of the procedures chart designed by the American Board of Forensic Odontology (ABFO) – Dental Age Estimation Committee. To conclude the operation in the software application, a Table for tooth-specific stage allocation was created and the estimated age was searched from the column of potential age matches. In the presence of two possibilities of age match, the youngest estimate was considered. If three possibilities were presented, the intermediate value was selected.

-Reproducibility

The main observer was a Forensic Odontologist with five years of experience as an official expert of the scientific police. In order to assess consistency during image analysis, an intraobserver agreement test was performed (re)assessing (T2) 10% (randomly selected subsample) of the main sample within 30 days from the main analysis (T1). After a subsequent period of 30 days, an additional intraobserver (re)assessment (T3) of another subsample (10%) was accomplished.

In parallel, a second observer was recruited to assess the same panoramic radiographs used in T2 to enable an interobserver agreement test. The rationale behind the number of radiographs re-assessed for examiner agreement tests was based on a previous dental age estimation study (12). Intra- and interobserver agreement tests compared continuous data (namely estimated ages in T1 and T2) and were quantified by means of Intraclass Correlation Coefficient.

-Statistics

Data treatment included descriptive statistics of central tendency and dispersion, such as minimum, maximum, mean, and standard deviation (SD) of chronological and estimated ages. Absolute (n) and relative (%) frequencies of distribution were used. The error of the method was presented as the difference between the estimated age and the chronological age, and was expressed as mean error, mean absolute error, and root mean squared error. Lin’s coefficient of concordance was used to test the distance between estimated and chronological ages. Visual representation of the difference between chronological and estimated ages was accomplished by means of Bland-Altman plots. Statistical significance was set at 5%, and confidence interval values within 95%. R software package was used (The R Foundation, Vienna, Austria).

## Results

In T1 and T2, the intraobserver reproducibility outcomes were 0.98 and 0.99 (95% confidence interval: 0.97; 0.99 in T1, and 0.98; 0.99 in T2). Interobserver reproducibility test outcome was 0.98 (95% confidence interval: 0.97; 0.99). The mean chronological and estimated ages of the combined sample were 10.88 ± 2.84 and 11.46 ± 3.21 years, respectively. In females (n = 509), the mean chronological and estimated ages were 10.95 ± 2.84 and 11.50 ± 3.05 years, respectively. In males (n = 595), the mean chronological and estimated ages were 10.83 ± 2.85 and 11.43 ± 3.35 years, respectively (Table 2). The mean errors between estimated and chronological ages were 0.56 and 0.60 years for females and males, respectively.

**Table 2:**
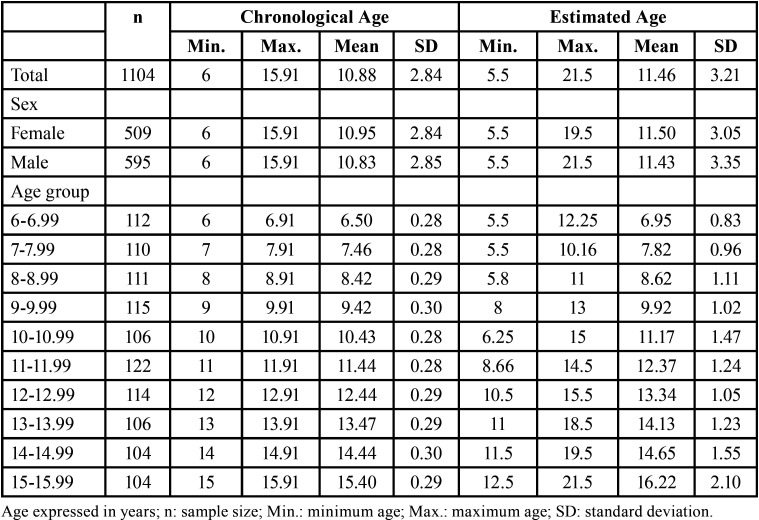
Descriptive statistics of the chronological and estimated ages of the sample distributed based on sex and age group.

**Figure 2:**
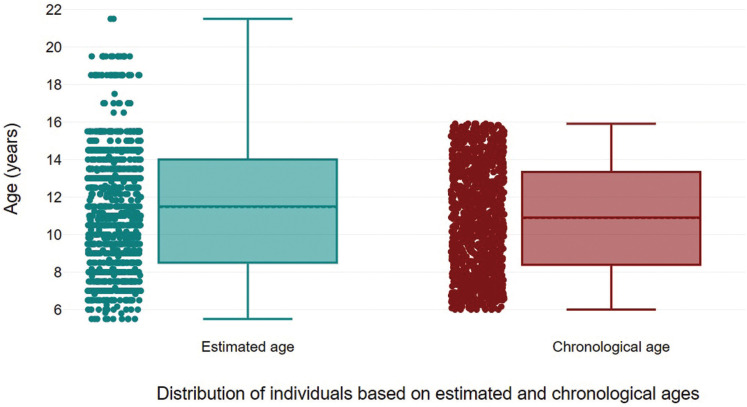
Distribution of individuals based on their chronological age and estimated ages. The red boxplot indicates a distribution preestablished based on the upper and lower limits of the chronological age set for sample collection (6-15.99 years), while the green boxplot highlights the dispersion of individuals, especially towards the upper age limit of the sample (indicating overestimations).

**Figure 3:**
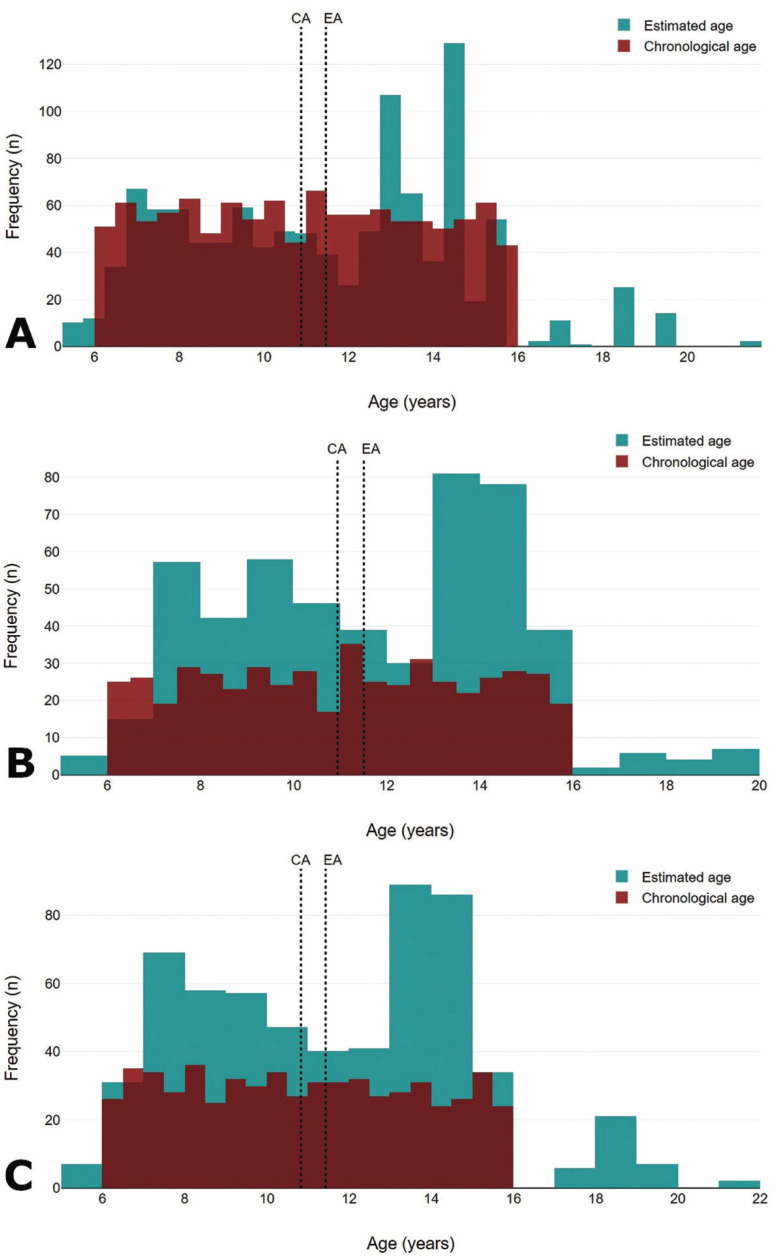
Frequency of individuals based on estimated (green) and chronological (red) ages. Estimated (EA, green) and chronological (CA, red) ages were superimposed, and the number of individuals distributed over the age (x-axis). Overestimations were prevalent throughout the age categories, but were more pronounced in older age groups of the combined sample (A), females (B) and males (C).

The best performances of the method were detected in the first four age groups (from 6 to 9.99 years) (Fig. 2), in which the values of mean absolute error varied from 0.72 to 0.92 years in females and from 0.59 to 0.90 years in males (Table 3). In all the age groups, the difference between estimated and chronological ages indicated a predominance of overestimations (Fig. 3).

**Table 3:**
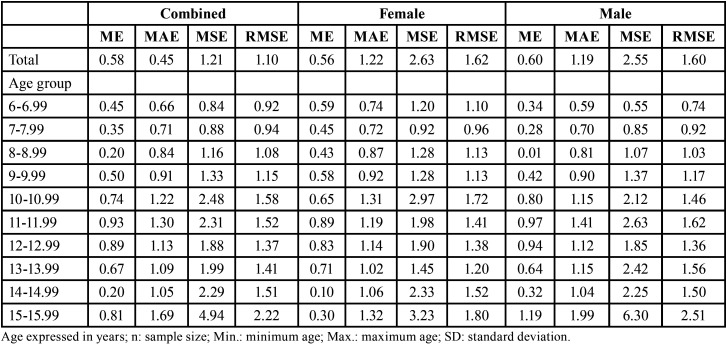
Descriptive statistics of the chronological and estimated ages of the sample distributed based on sex and age group.

**Figure 4:**
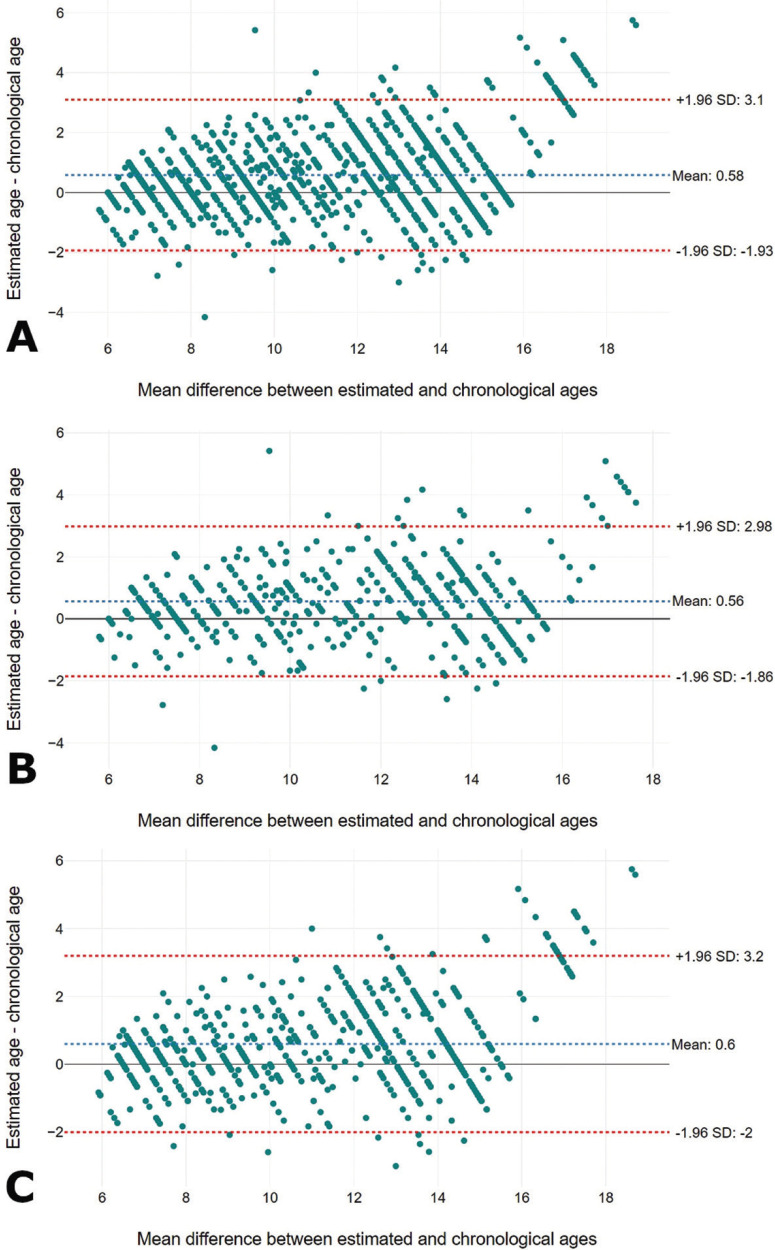
Bias of the method represented by the difference between estimated and chronological ages. The overall bias observed in this study was 0.58 years for the combined sex (A), and 0.56 years for females (B) and 0.60 for males (C). For the three studied sex categories, the bias was represented by an overestimation of was almost of 7 months. Between estimated and chronological ages.

The overall Lin’s coefficient of concordance was 0.9 for the combined sex category, and for females and males (*p* > 0.05), separately (Table 4) – showing moderate agreement between estimated and chronological ages. Visualization of the difference between estimated and chronological ages is presented with scatter plots that show a mean bias of the method of 0.58 years for the studied population (females = 0.56 years, males = 0.60 years), (Fig. 4).

**Table 4:**
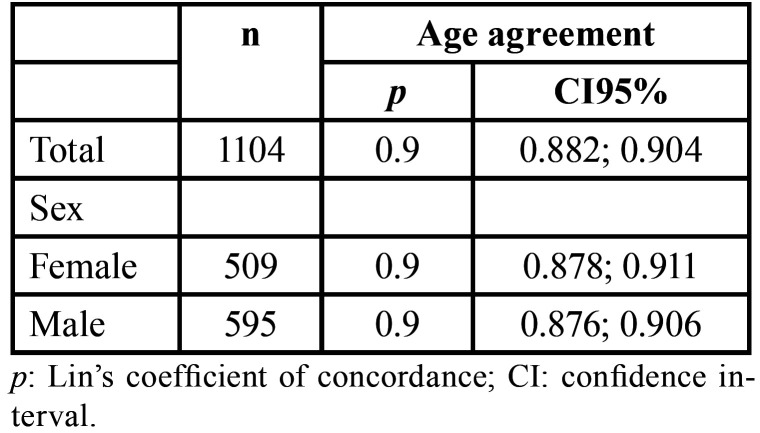
Measures of concordance between estimated and chronological ages.

## Discussion

Dental age estimation has contributed to clinical and forensic practices (23). Since the original publication of The London Atlas in form of a scientific article in 2010 (1), several population-specific validation studies have corroborated the applicability of the method. In the scientific literature, there are two main strategies to test the applicability of the London Atlas: by assessing the deciduous and permanent dentition among individuals below the age of 16 years (24), and by assessing the development of third molars among individuals between 16 and 23 years (4). In children, the applicability of the London Atlas is known for its credibility. E.g. Ismail *et al*. (24) observed that the method did not present statistically significant differences for age estimation in late childhood compared to other traditional methods. In adolescents, on the other hand, the application of the method remains encouraged, but with caution (5). The present study tested the applicability of the London Atlas Software App 2nd edition for age estimation of Brazilian children, using not only the mandibular (permanent) teeth usually assessed in children, but the third molar of the same quadrant. This methodological decision is worth discussing because the inclusion of an additional tooth could lead to a more comprehensive assessment of age. However, third molars are known for the high variability and could drag down the performance of the method.

Our overall outcomes demonstrated mean error rates from 6.7 to 7.2 months of difference between estimated and chronological ages. Compared to the outcomes of other methods applied to Brazilian children and pooled in a recent meta-analysis (15), the present study’s outcomes could be considered acceptable for clinical practice, and potentially relevant to the forensic field. What must be noted, however, is the increasing mean absolute error over time. The mean absolute error expresses the difference between a true value (known/chronological age) and a measured value (estimated age) without considering the direction of the error (positive/negative or over-/underestimation). In this study, the mean absolute error for the combined sex group did not exceed 0.91 years in the age interval between 6 and 9.99 years. In the remaining age intervals (10-15.99 year), however, the mean absolute error was between 1.05 and 1.69 years. The improved error rates in early childhood can be justified by the several teeth developing simultaneously. In older age groups, more teeth with complete apex closure will appear and the third molar development will be more decisive in the age estimation process. In this context, Sousa *et al*. (5) warned about the application of the London Atlas when third molars are used as decisive teeth. The authors also found mean absolute errors above 1.07 years in late childhood and advised caution for forensic applications when the other permanent teeth are not developing.

Knowing the error rates of methods used by experts is one of the factors considered for the admissibility of forensic evidence (26). The current study presents error metrics quantified to clarify on the performance of the method. Hence, the clinical application of the London Atlas can be defended depending on the treatment. In the forensic field, the method can contribute to the age estimation of children. According to the American Board of Forensic Odontology (https://abfo.org), age estimation of children should consider “the radiographic evaluation to stage the degree of morphologic development of the primary and/or secondary dentition as well as resorption of the primary dentition. Infant/Child techniques should consider sex, ancestry, and population specificity”. Thus, third molars could be reserved for dental age estimation in late adolescence – when third molars are the only developing teeth.

Previous dental age estimation studies that considered the population specificity and method validation (factor addressed by the ABFO) revealed much lower error rates in Brazilian children for methods that were solely based on permanent teeth (except third molars) (12,15). Thus, it can be estimated that the inclusion of third molars together with other teeth to assess the age of children with London Atlas does not necessarily improve the error rates for specific age categories. Similar results have been observed with other methods as well. For instance, Franco *et al*. (12) found in a study with Willems’ method (25) that the combination of the development of third molars and other permanent teeth could improve age estimates only discretely and only for females in early adolescence (14-15 years). Future studies in the field are encouraged to test age estimation with (multivariate analysis) and without third molars, and addressing other geographic populations of Brazil to promote a more comprehensive and general understanding of the performance of London Atlas Software App 2nd edition in the country. Similarly, global analyses of the performance of the method are encouraged by means of systematic reviews and meta-analyses.
